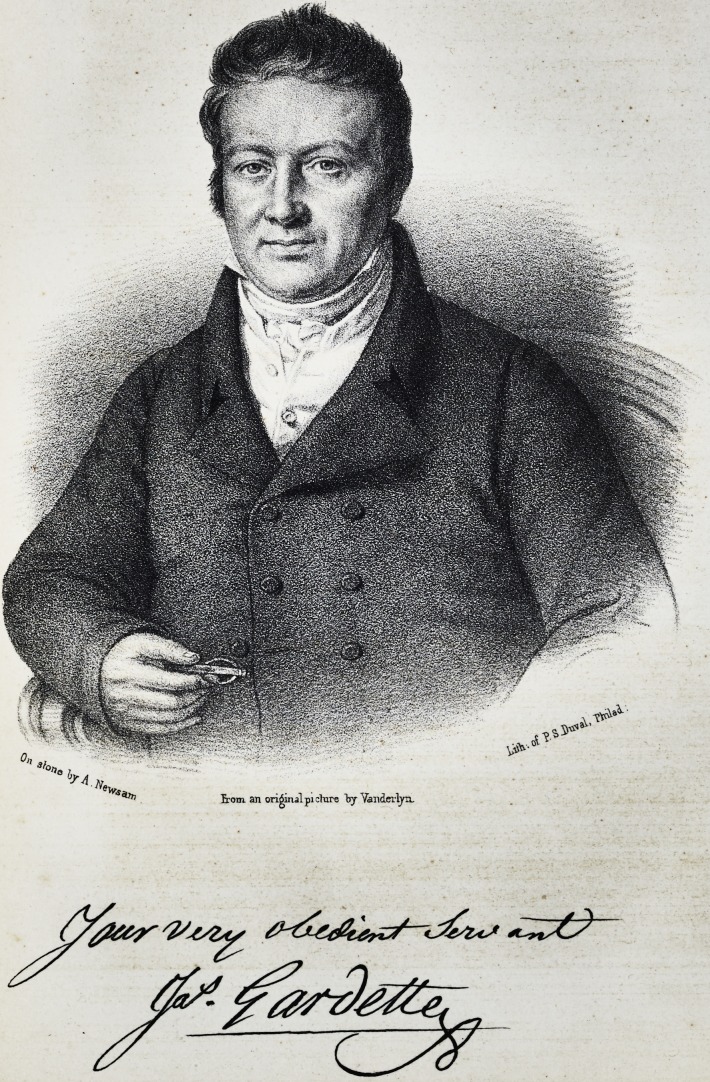# Biographical Notice of the Late Dr. James Gardette, Surgeon Dentist, of Philadelphia

**Published:** 1851-04

**Authors:** Emile B. Gardette

**Affiliations:** Dentist.


### BIOGRAPHICAL DEPARTMENT.

*Biographical Notice of the late* James Gardette, *Surgeon Dentist, of Philadelphia.* By Emile B. Gardette, M. D., Dentist.

James Gardette, Surgeon Dentist, was the second son of *Jean Blaize* Gardette, and was born 13th of August, 1756, in the town of *Agen, département de Lot et Garonne*, France. His father died when James was quite a lad, and we are but little acquainted with this early period of his life; nor, indeed, does it enter into the plan for the performance of our task. We only know that he possessed a very trifling patrimony, insufficient for his maintenance or education, and that after his father's death he was brought up by his paternal uncle, *Blaize* Gardette, who lived at Agen, and held the office of prosecuting attorney until an advanced age. His uncle designed James for the medical profession, and with that view, after the ordinary academical studies of that day in a provincial town of France, sent him to Paris. He remained at the capital about two years, (from 1773 to 1775,) pursuing the study of anatomy and surgery in the Royal Medical School; and thence he was removed to the Hospital at *Toulouse*, where he resided eighteen months, as a pupil in the institution. At the end of this period he was sent to *Bayonne*, and there was examined by the surgeons of the Admiralty, and commissioned as a surgeon in the French navy.

We are not aware whether this first step in life, or the more important one that immediately followed it, met the approbation of the good old uncle at *Agen;* but the probability is that they were the voluntary independent movements of the young and more ambitious nephew. For we find that immediately on obtaining the commission in the navy, he received orders to embark, in his professional capacity, on board the brig of war

*La Barquaize de St. Jean de Luz*, destined for Boston, Massachusetts. He sailed in October, 1777, and arrived at Plymouth early in January following.

The love of liberty and popular movement throughout France, which brought so many young Frenchmen to the United States at the period of our "Declaration of Independence," had no small influence in governing the course of Mr. Gardette. He made a cruise of four months, during which an engagement occurred with two British ships, lasting three hours and a half, and in which there were several killed and wounded on board the vessel of which he was the surgeon. This seems to have terminated his official duties and connection with the French navy, from which he resigned, intending to adopt this country as his home. When the French fleet and army arrived at Newport, he was induced to visit that town, and commence practice as a dentist, the officers affording him congenial and considerable occupation for a short time. He had received instructions in dental operations, (as part of his profession of naval surgeon,) from Mr. Le Roy de la Faudinière, a dentist at Paris, then in high repute. He had also provided himself with the best works extant, (Fauchard and Bourdet,) on the teeth, and with a limited set of dental instruments: still we scarcely think he could have had any expectations of pursuing the profession of dentist in this country at the time he left France.

He returned to Boston from Newport, and in the autumn of 1783, we find, went to New York. He was there when the American army, under General Knox, took possession of the city—an inactive but not indifferent spectator of the great events of that interesting epoch in American history.

It is worthy of remark, (and the thought may not be out of place here,) that the French emigrants who selected this as the country of their adoption, from sympathy with its institutions, have proved to be both useful and creditable citizens: they rarely, if ever, sought to fill public offices, or to meddle with politics beyond the fair expression of their sentiments, and the conscientious exercise of the elective franchise when justly entitled to it.

As a class, the French naturalized citizens have afforded strikingly correct examples of temperance and propriety—an upright, unobtrusive and industrious population, which we think, and may be permitted to say, has not been duly or justly estimated in a public or political sense. The first cause that operates towards this result is, no doubt, the fact of not speaking the language of the country, and, even when acquired for all useful purposes, the pronunciation, in most cases, cannot be overcome; and thus Frenchmen of learning and high integrity of character, after long residence among us, have not commanded the general respect and influence to which they were justly entitled.

Mr. Gardette's professional success as a dentist in New York seems to have been comparatively small, and his limited knowledge of the English language was, as yet, a great impediment to making himself known or appreciated as he desired. It was not until the summer of 1784, and in Philadelphia, that he attained the position which determined his permanent residence in the United States. The pleasant and successful character of his occupation among the best class of citizens in Philadelphia, at the period when Fourth street was its western boundary, needs, perhaps, no stronger comment than the fact that *he continued there, in excellent uninterrupted practice as a dentist, from* 1784 *to* 1830—*a period of forty-six years!**

* In Mr. E. Parmly's Appendix to "Dentalogia," a Poem by Mr. S. Brown, we find a republication of an Obituary Notice of the late Dr. Ed. Hudson, dentist, which originally appeared in a city newspaper, under the signature of "A Surgeon Dentist," and which, Mr. Parmly tells us, is from the pen of Dr. S. S. Fitch. We feel that it would be an act of injustice to the professional character of James Gardette, to omit noticing here an article calculated to mislead the reader and disguise the truth; deriving importance chiefly, it is true, from having obtained space in a volume which bears the name of so distinguished a dentist and estimable a man as Mr. E. Parmly.

*Extract from the "Appendix."*—"When he, (Dr. Hudson,) commenced his practice here, he found the profession, generally, at a *very low ebb*—usually exercised by mechanics. Those great principles which elevate dental surgery from an art to a science were *almost entirely overlooked or*

Among the eminent physicians of that period, Drs. Wistar, Shippen, Kuhn and Rush, befriended and encouraged him by such aid and courtesy as were due to his correct professional views, and his education and manners as a gentleman—characteristics which, we may safely conclude, were not very commonly found among the *soi-disant* dentists of our country at that remote day. Mr. Gardette devoted himself attentively to the pursuit and improvement of his profession, and acquired no unenviable reputation for knowledge and skill in its various departments.

The difficulties which the dentist then had to contend with were manifold: he was dependent chiefly upon his own judgment and inventive genius for his success, and that, too, for the benefit of patients who, in many instances, had but little confidence in the operations of dentistry. Instruments were very defective, and not to be had in this country; and even the ma-

*unknown.* To *remove this mass of rubbish,* to obliterate bitter and widely extended prejudices, was the task of Dr. Hudson," &c., &c.

Other passages are scarcely less objectionable on the score of impartial truth—for Dr. S. S. Fitch cannot well have been ignorant that when Dr. Hudson commenced his practice here in Philadelphia, (about 1805–6,) *he had never to any extent practiced anywhere else;* and that Mr. Gardette had already, as a practitioner of about twenty years' standing, acquired and deserved a high reputation for science and skill in his profession. Dr. Hudson himself was among those who acknowledged and honored that reputation, and in cases of doubt, in his own early practice, he sought the benefit of Mr. Gardette's experience. It would have been but a slight effort of justice and truth to have excluded Mr. Gardette from the "*mass of rubbish*" which it became the task of Dr. Hudson to clear away; and to have extended the same just exception in reference to the "very low ebb" at which the profession stood in Philadelphia "when Dr. Hudson commenced practice here." The exalted professional position ascribed to Dr. Hudson was justly his due at a later period of his life, when he attained merited distinction, spite of accumulated "rubbish:" we are ready and glad to name him as among the best educated and most successful dentists of modern times.

Had Dr. S. S. Fitch's article possessed no other than the passing existence afforded by a newspaper, it had probably never claimed notice here, but been allowed all the honor that belongs to *undeserved and uncontradicted misrepresentation.*

terials which were recognized as appropriate for professional use could not be obtained short of Paris or London. Among the improvements introduced into the practice of dental surgery by Mr. Gardette, whether in the way of instruments or operations, some few, at least, have been identified with his name; and we cannot better show the estimate placed upon them than by the following extract from the Minutes of the "*John Scott Legacy for the Encouragement of Useful Inventions in the Arts and Sciences.*"

"1822—To James Gardette, dentist, for three mechanical improvements in his profession, which are highly commended in Europe and in the United states; and for a simple lever instrument for the easy and expeditious extraction of teeth and stumps of teeth, awarded a medal 'to the most deserving,' and twenty dollars."

The above "award of merit" is the highest permitted by the will of John Scott, who left the fund, (secured, we believe, to the city in trust,) for the objects specified.

This brief, and, (as regards the nature of the "mechanical improvement,") unsatisfactory account is all we are able to discover from the archives transferred into the hands of the Franklin Institute. But we think we can enumerate most of the inventions which the profession owes to Mr. Gardette, without injustice to others.

He was the first dentist who substituted the use of elastic flat gold bands or braces, in the place of ligatures of silk or fine gold wire, for securing artificial teeth when attached to the living ones.*

* Mr. L. Laforgue, a distinguished dentist and writer of Paris, says, in his "*Théorie et Pratique de l'Art du Dentiste,*" 2d edition, 1810, p. 20—

*Translation.*—"The plan of maintaining artificial teeth by means of ligatures is almost entirely done away with by Gardette of Philadelphia: he secures artificial pieces without tying them, even when of limited extent. I have seen such admirably secured, and am acquainted with no dentist who equals him in this beautiful and valuable description of work."

In pp. 257–294, Laforgue refers to the invention of gold mortise plates, for mounting artificial teeth, as due to Gardette, of Philadelphia.

He invented the manner of mounting natural teeth, which consists of a gold mortise plate, to which the teeth are secured by means of gold pins, and which permits the *tooth* to rest upon the gum instead of the gold plate.

He was the first to apply the principle of *suction* or *atmospheric pressure** for the support of entire sets of artificial teeth, dispensing with the use of spiral springs and the endless contrivances then in use, much to the inconvenience of those who wore them.

Nor were his improvements less important in the cure of diseases to which the teeth and gums are liable: he was the early advocate, if not the first who recognized the wisdom, of affording space for the healthy and good arrangement of the teeth by judicious extractions in youth. He believed, and his

*It is a well-authenticated fact, that Mr. Gardette successfully maintained sets of artificial teeth for the upper jaw, on the principle of atmospheric pressure, as early as 1800.

We have heard him relate the following anecdote of the chance which led to this important discovery. He had furnished, for the second time, an entire set of upper teeth, (enameled hipp[illegible],) for Mrs. A. M'C., and owing to the short time the first set had lasted, under the action of the saliva, he suggested that this set should be left much heavier. In order that the tongue should become accustomed to this increased bulk, necessarily contracting the limits for its free movements, the lady was desired to keep the new piece in her mouth as much as possible, during a few weeks, but not expecting her to use it for purposes of mastication or speech until the usual springs should be attached to it. Mr. G. promised, at the end of the period named, to call and arrange the piece for permanent use.

It was then still the custom for the dentist to attend at the houses of his patients, and a busy season caused months instead of weeks to elapse, when Mr. Gardette called again: with an apology for neglect, his plyers and springs ready, he requtrted Mrs. M'C. to bring the artificial pieces. She replied, "I have them in my mouth," much to the astonishment of her dentist, with whom she had been conversing with her usual facility. She stated that at first they were a little troublesome, but she had become accustomed to them now, and they answered every purpose as well *without* as *with* springs, and she was glad to dispense with them. The principle upon which the artificial piece thus adhered to the gum, at once suggested itself to his mind, and suction, or atmospheric pressure, was henceforth depended upon in numerous cases of the same kind.

long experience proved, that he thus obviated a great cause of decay, arising from lateral pressure, when the circle of the jaw is too narrow for the number and size of the teeth to permit their regular and easy arrangement.

He was one of the earliest dentists who adopted *gold foil*, instead of *lead* or *tin*, as the best material for filling teeth; and related often that he had at one period prepared gold foil for his own use from Dutch ducats, when no gold-beater was to be found in this country, or none, at any rate, who could furnish dentist's filling gold.

As an *operator*, Mr. Gardette displayed great judgment, care and dexterity, while he exhibited no misplaced or morbid sensibility inconsistent with the best performance of his painful professional duties.

In the mechanical departments of his art, his work evinced discrimination and good taste, as well as originality: his artificial pieces, at a period when no aid was to be derived from "*dental laboratories*," possessed all the good workmanship and finish which are the result of mechanical skill and patient industry.

His practice was characterized by the one strong motive of *good to his patient*, and not less by the liberal and benevolent feelings which should govern professional life.

His want of familiarity with the English language seems to have made him diffident about publishing his views or improvements in his profession; and it was not until 1827 that he was induced by his friend, the late Dr. James Mease, (a liberal and warm friend of the arts and sciences,) to furnish an article for the "Medical Recorder," on the "Transplantation of the Human Teeth;" the first, and, we believe, the only publication that bears his name. This paper, occupying seven pages of the periodical referred to, (January, 1827,) goes to show the impracticability and injudicious character of the operation, and exhibits a sound and sensible theory, with some original suggestions.

As a practicing dentist, the usefulness of Mr. Gardette was much impaired, during the latter years of his life, by continued and severe suffering from the gout. He had long cherished a desire to return to France, and end his days in his native country, but owing to unfortunate investments and various disappointments, this favorite plan was not accomplished until the year 1829, at the age of seventy-three, too late to realize the pleasant anticipations he had so long connected with such a step. His native village of Agen, which he revisited, was no longer what it had seemed to his longing heart, during an absence of half a century: he took up his residence at Bordeaux, where he did not attain his expected contentment, and had already, in his letters, expressed thoughts of returning to this country. But before any such design could be carried out, he was attacked by his old enemy, the gout, and died in August, 1831.

---

## Figures and Tables

**Figure f1:**